# Design and optimization of ethanol production from bagasse pith hydrolysate by a thermotolerant yeast *Kluyveromyces* sp. IIPE453 using response surface methodology

**DOI:** 10.1186/2193-1801-2-159

**Published:** 2013-04-15

**Authors:** Diptarka Dasgupta, Sunil Kumar Suman, Diwakar Pandey, Debashish Ghosh, Rashmi Khan, Deepti Agrawal, Rakesh Kumar Jain, Vasanta Thakur Vadde, Dilip K Adhikari

**Affiliations:** Biotechnology Conversion Area, CSIR-Indian Institute of Petroleum, Mohkampur, Dehradun, UK 248005 India; Division of Chemical Recovery, Central Pulp and Paper Research Institute, Biotechnology & Lignin by-products, Saharanpur, UP 247001 India

**Keywords:** Thermotolerant yeast, *Kluyveromyces* sp. IIPE453, Sugarcane bagasse pith, Response surface method

## Abstract

Ethanol production from sugarcane bagasse pith hydrolysate by thermotolerant yeast *Kluyveromyces* sp. IIPE453 was analyzed using response surface methodology. Variables such as Substrate Concentration, pH, fermentation time and Na_2_HPO_4_ concentration were found to influence ethanol production significantly. In a batch fermentation, optimization of key process variables resulted in maximum ethanol concentration of 17.44 g/L which was 88% of the theoretical with specific productivity of 0.36 g/L/h.

## Introduction

The global scenario demonstrates that lion share of research in past three decades have been focused on technological know-how development for bioethanol since its emergence as a potential fuel additive. All the key challenges on energy and economic front have already been pinpointed in various forums as sole restrictors for commercialization of lignocellulosic bioethanol technology ( Cardona et al. 2010 ). Evidently a non-molasses feedstock was to be brought into reality to meet excess ethanol demand for 5-10% compulsory blending. Biomass being a cheap and renewable raw material with abundant availability ( Saxena et al. 2009 ; Kumar et al. 2009a ; Cheng et al. 2008 ), has been considered as an excellent feedstock for bioethanol production due to its high holocellulosic content.

Ethanol production via fermentation route comprises of a series of biochemical reactions with numerous factors involved in the process. Conversion of lignocellulosic sugar hydrolysate into ethanol requires many other micro and macro elements apart from fermentable nitrogen which in right balance can always give optimum product yield. Statistical screening in this context provides a rapid assessment of key process variables in a systematic way whereby a perfect strategy can be materialized to improve targeted product yield. Response surface methodology (RSM) explores the relationships between several explanatory operating variables and one or more response variables and has been widely applied for optimization of ethanol production from various substrates ( Uncu & Cekmecelioglu 2011 ; Jargalsaikhan & Saraçoğlu 2009 ).

In this paper, we have carried out RSM study of ethanol fermentation with thermotolerant yeast *Kluyveromyces* sp. IIPE453 (MTCC 5314) (Kumar et al. 2009b ) to find optimum conditions for maximizing ethanol production via two step approach. Initial screening of factors were performed with Plackett-Burman Design (PBD) method to identify crucial parameters ( Dong et al. 2012 ; Maruthai et al. 2012 ) affecting ethanol yield and to the degree based on their individual effect and interactions through Box-Behnken Design (BBD) technique ( Mei et al. 2011 ; Palukurty et al. 2008 ). Further, an optimization study was conducted to maximize ethanol yield in shake flask. Optimized data has also been evaluated at bench scale bioreactor of 2 L working volume. One of the unique characteristics of *Kluyveromyces* sp. IIPE453 is its ability in utilizing pentose sugar for growth and fermentation with hexose sugar. Yeast cell biomass was grown with pentose rich fractions obtained after acid pretreatment of sugarcane bagasse (SCB) pith and fermented with glucose rich broth obtained after enzymatic saccharification of the pretreated pith.

An average sugarcane bagasse contains 35% pith and with 60% depithing efficiency, around 20% pith is removed during depithing operation either at sugar mill site or at paper mill premises. An average 300 tpd (Tonne per day) bagasse based paper mill generates 160 tpd pith ( Jain et al. 2011 ). Pretreated pith has been utilized as substrate for production of single cell proteins ( Rodriguez-Vazquez et al. 1992 ) as well for preparation of activated carbon for dye removal from aqueous solutions ( Amin 2008 ). However, utilization vs. generation ratio is almost negligible. Even after using as a boiler fuel in paper industry itself (calorific value of 17.07 Kcal/Kg ( Diez et al. 2011 )) huge amount of pith remains unutilized and poses serious waste disposal problem. Bagasse based paper mills in India annually generates 45 – 55 million tons pith with a biochemical composition of holocellulose (68–69% w/w) including hemicellulose (20-21% w/w), lignin (21–22% w/w) and ash (6–7% w/w) ( Sanjuan et al. 2001 ) which can be effectively used for ethanol production and thereby value addition to waste.

This study was conducted based on using this feedstock in order to integrate the process in a bio refinery mode attached with a sugar or paper and pulp industry and probably the first paper of this kind to the best of our knowledge.

## Materials and methods

### Materials

Bagasse pith sample was generated in a depither unit with 100 mesh size at Central Pulp and Paper Research Institute (CPPRI), Saharanpur, Uttar Pradesh and used at CSIR-IIP, Dehradun for hydrolysis and saccharification. SCB pith was treated with steam and acid (8% w/w H_2_SO_4_) in a solid vs. liquid ratio of 1:10 at 120°C. for 90 minutes to extract pentose rich fraction (20 g/L) which was used as carbon source for cell biomass generation. Pretreated pith devoid of pentosans was further enzymatically saccharified using commercially available cellulase (Advanced Biochemicals Ltd, Mumbai, India) to get hexose rich stream (40 g/L) for ethanol fermentation. Saccharification was carried out using 7% w/w of enzyme with solid vs. liquid ratio of 1:10 at 50°C. for 22 h.

### Micro-organism and culture conditions

*Kluyveromyces* sp. IIPE453 (MTCC 5314), a thermophilic yeast (optimum growth temperature 45°C.) isolated from dumping sites of crushed SCB in a local sugar mill was used in this experimental study. The stock culture was maintained on YPD agar medium (composition in g/L; yeast extract, 10.0; peptone, 20.0; dextrose 20.0; *agar agar* 20.0; pH 4.5-5.0).

### Experimental design

Growth was carried out in prehydrolysate (pentose broth) at 45°C. for 16 h. Nutrient screening and optimization for ethanol production were performed at same temperature in shake flasks (80 ml working volume) in the hydrolysate (hexose broth) supplemented with various nutrients according to experimental design. The physical parameters, pH and fermentation time were maintained as per design specifications (Table 1). All experiments were carried out in replicates and results are reported in terms of mean values. Experimental design and statistical analyses were done using *Reliasoft* Design of Experiment (DOE) software with risk factor (α) values of 0.05 (95% level of confidence) for PBD and 0.01 (99% level of confidence) for BBD. Criterion of predicted model acceptance was based on their adjusted coefficient of regression (R_adj_^2^) with value above 0.95. Variables with P values lower than 0.05 (PBD) and 0.01 (BBD) were considered to have significant effect on the response.

**Table 1:** **Plackett burman design for screening of factors**

Serial	Run order	pH	Inoculum volume (%v/v)	Substrate conc. (g/L)	Yeast extract (g/L)	MgSO_4_ (g/L)	(NH_4_)_2_SO_4_ (g/L)	Na_2_HPO_4_ (g/L)	KH_2_PO_4_ (g/L)	Fermentation time (h)	Response variable ethanol conc. (g/L)	
											Experimental	Model predicted
1	1	+1	+1	-1	+1	+1	+1	-1	-1	-1	7.20	6.91
2	6	-1	-1	-1	+1	-1	+1	+1	-1	+1	8.05	8.14
3	3	+1	-1	+1	+1	-1	+1	+1	+1	-1	7.18	7.18
4	2	-1	+1	+1	-1	+1	+1	+1	-1	-1	8.07	8.35
5	4	-1	+1	-1	+1	+1	-1	+1	+1	+1	7.86	7.76
6	10	-1	+1	+1	+1	-1	-1	-1	+1	-1	9.03	9.13
7	11	+1	-1	+1	+1	+1	-1	-1	-1	+1	10.09	10.37
8	8	+1	+1	-1	-1	-1	+1	-1	+1	+1	8.05	8.33
9	9	+1	+1	+1	-1	-1	-1	+1	-1	+1	7.30	7.02
10	12	-1	-1	-1	-1	-1	-1	-1	-1	-1	8.20	8.10
11	5	-1	-1	+1	-1	+1	+1	-1	+1	+1	13.40	13.12
12	7	+1	-1	-1	-1	+1	-1	+1	+1	-1	5.60	5.69

**Table 2:** **Factors with their coded levels**

Serial number	Variable	Low (-1)	Center point (0)	High (+1)
1	pH	4.5	5	5.5
2	Fermentation time (h)	24	36	48
3	Substrate Concentration (g/L)	20	30	40
4	Yeast extract (g/L)	1	2.5	5
5	MgSO_4_(g/L)	0.06	0.09	0.12
6	(NH_4_)_2_SO_4_ (g/L)	1	3	5
7	Na_2_HPO_4_(g/L)	0.15	0.30	0.45
8	KH_2_PO_4_(g/L)	0.15	0.30	0.45
9	Inoculum volume (% v/v)	5	7.5	10

#### Plackett burman design

A two level PBD ( Plackett & Burman 1946 ) experimental matrix was set up to identify the factors and estimate their significance in ethanol production. It predicts linear model where only main effects are taken into consideration.

**Table 3:** **Box behnken design**

Run order	Random	Substrate conc. (g/L) (A)	pH (B)	Fermentation time (h) (C)	Na_2_HPO_4_ (g/L) (D)	Experimental ethanol conc. (g/L)	Model predicted ethanol conc. (g/L)
1	15	0	-1	0	+1	10.90	10.32
2	14	0	+1	0	-1	8.60	7.79
3	9	-1	0	-1	0	4.20	3.95
4	12	+1	0	+1	0	11.55	12.07
5	4	+1	+1	0	0	7.10	6.91
6	17	-1	0	0	-1	9.30	9.34
7	19	-1	0	0	+1	4.40	4.35
8	20	+1	0	0	+1	11.50	11.47
9	24	0	+1	+1	0	7.90	7.45
10	7	0	0	-1	+1	7.25	7.37
11	25	0	0	0	0	7.60	8.01
12	11	0	0	+1	0	8.32	7.65
13	2	+1	-1	0	0	14.60	13.53
14	13	0	-1	0	-1	12.70	12.61
15	8	0	0	+1	+1	8.00	8.45
16	10	+1	0	-1	0	8.10	8.37
17	23	0	-1	+1	0	12.00	12.27
18	3	-1	+1	0	0	3.60	4.29
19	1	-1	-1	0	0	7.50	7.31
20	21	0	-1	-1	0	7.90	8.57
21	5	0	0	-1	-1	6.80	7.04
22	6	0	0	+1	-1	12.80	13.36
23	18	+1	0	0	-1	11.00	11.06
24	26	0	0	0	0	8.00	8.01
25	16	0	+1	0	+1	5.40	5.50
26	27	0	0	0	0	7.70	8.01
27	22	0	+1	-1	0	4.10	3.75

1

Response indicates dependent variable in terms of overall ethanol production (g/L), **a** being the model intercept. **X**_**i**_ represents different levels of independent variables with **b**_**i**_ coefficients as predicted by the equation. In this paper, 9 independent variables were selected, e.g. physical parameters such as pH, fermentation time, inoculum volume (%v/v) and media components such as sugar concentration, yeast extract, magnesium sulphate [MgSO_4_], ammonium sulphate [(NH_4_)_2_SO_4_], di-sodium hydrogen phosphate [Na_2_HPO_4_] and potassium di-hydrogen phosphate [KH_2_PO_4_]. Table 1 illustrates the design matrix of various components with coded values; low (-1) and high (+1) while Table 2 represents their actual values. *Pareto* charts were plotted to highlight most significant factors responsible for ethanol production.

#### BBD design and optimization

BBD technique is a statistical indication of quadratic effect factors obtained after initial factorial screening studies and their interactions ( Box & Behnken 1960 ). Based on *Pareto* chart results, BBD matrix was constructed with four significant factors (substrate concentration, pH, fermentation time and Na_2_HPO_4_ concentration) each having 3 levels (-1, 0 and 1) with 27 experimental designs as shown in Table 3. Rest non-significant factors namely Inoculum volume, Yeast extract, MgSO_4_, (NH_4_)_2_SO_4_ and KH_2_PO_4_ were maintained at their respective low level values (Table 2). A second order polynomial model was predicted with DOE (equation 2) indicating linear, interaction and quadratic effect of variables on system response as either + ve or -ve. ANOVA analysis of the model was performed to evaluate its statistical significance.2

where, A, B, C and D are the independent variables, a_1_ is an offset term, b_1_ to b_4_ are linear term coefficients, b_5_ to b_10_ indicate interaction terms and b_11_ to b_14_ represent quadratic effect.

**Figure 1:**
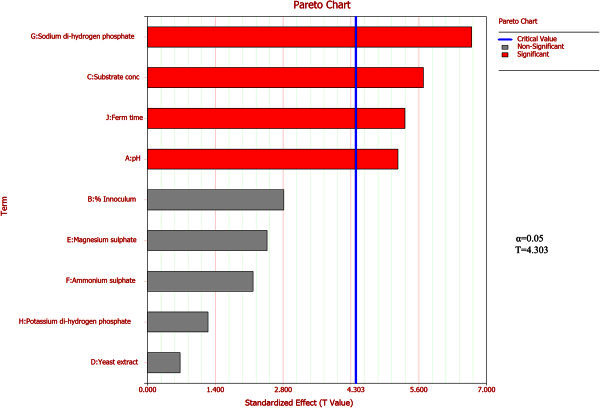
**Pareto chart of Placket Burman design.**

**Table 4:** **Regression analysis for Plackett Burman design variables**

Term	Effect	Coefficient	Standard error	T value	P value
**Intercept**		**8.3376**	**0.1481**	**56.296**	**0.0003**
**pH**	**-1.5322**	**-0.7661**	**0.1481**	**-5.1726**	**0.0354**
% Inoculum (v/v)	-0.8345	-0.4173	0.1481	-2.8174	0.1063
**Substrate conc.**	**1.6883**	**0.8441**	**0.1481**	**5.6997**	**0.0294**
Yeast extract	-0.1995	-0.0997	0.1481	-0.6734	0.5701
Magnesium sulphate	0.7329	0.3665	0.1481	2.4744	0.1318
Ammonium sulphate	0.6458	0.3229	0.1481	2.1803	0.161
**Sodium di-hydrogen phosphate**	**-1.984**	**-0.992**	**0.1481**	**-6.6982**	**0.0216**
Potassium di-hydrogen phosphate	0.3705	0.1853	0.1481	1.251	0.3374
**Fermentation time**	**1.5751**	**0.7875**	**0.1481**	**5.3174**	**0.0336**

### Model validation in shake flask and scale up study in bench scale bioreactor

BBD predicted response led to identification of optimization conditions in terms of key independent variables having significant effect on system response. To validate authenticity of software generated model, fermentation was carried out in shake flask under optimized conditions and further tested on a 2 L bench scale bioreactor (NBS Bioflo 110) equipped with *supervisory control and data acquisition* (SCADA) system. Yeast biomass generated on prehydrolysate (20 g/L pentose conc.) was inoculated in fermentation broth having SCB pith hydrolysate (40 g/L glucose conc.) in shake flask as per model predicted optimized conditions as well as in 2 L NBS bioflo110.

### Analytical methods

Sugar and ethanol concentration (g/L) was quantified by HPLC (UFLC Shimadzu) with PL Hiplex-H acid 8 μm column (100 × 7.7 mm diameter, by PL Polymer laboratory, UK). The column was eluted with a mobile phase 1 mM sulfuric acid at a flow rate of 0.7 ml/min at column oven temperature 57°C. with RI detector.

## Results and discussion

### Evaluation of key variables affecting ethanol production

Lignocellulosic ethanol production requires various micro and macro elements along with fermentable sugar and nitrogen which in best commingle results in optimum product yield where a controlled environment is again a prerequisite ( Asli 2009 ; Anupama et al. 2010 ). Magnesium, being the cofactor for glycolytic enzymes involved in fermentation ( Lodolo et al. 2008 ) and potassium being the regulator of pH via K^+^/H^+^ transport system, ( Kudo et al. 1998 ) are essential cations governing ethanol fermentation. Ammonium salts stimulate glucose fermentation by lowering induction period ( Muntz 1947 ) and maintaining an optimum carbon to nitrogen (C/N) ratio. Substrate concentration primarily affects uptake rates and thereby product rate kinetics. High substrate concentration negatively hampers ethanol productivity leading to a lower titer due to repression of glycolytic enzymes ( Bisson & Fraenkel 1984 ). Yeast extract is a rich source of vitamins and promotes cell growth and proliferation. Hence, the above mentioned variables have been chosen to screen and develop a low cost fermentation medium with optimum blend of nutrients and physical parameters for bioethanol production.

PBD identified the key variables among selected ones via *Pareto* chart illustrated in Figure 1. Factors such as pH, fermentation time, substrate concentration and Na_2_HPO_4_ with T values above threshold (4.30 in this case) and P values lower than 0.05 as represented by regression analysis (Table 4) had a substantial effect on ethanol yield and were considered for further evaluation by BBD, while rest of the variables did not have a meaningful contribution to ethanol production. Fermentation process is directly affected by the amount of viable cells present in broth. An optimum inoculum volume of 5% (v/v) was sufficient to carry out the fermentation process. Higher concentration of the same had no effect on ethanol yield improvement and thus was considered to be non-significant variable in the process (Figure 1). The model considering main effects (equation not shown) was found to be fairly accurate having a R^2^ value of 0.98 with a R_adj_^2^ value of 0.92 with experimental and model predicted response being fairly close to each other.

**Table 5:** **ANOVA table for BBD model**

Source of variation	Degrees of freedom	Sum of squares [Partial]	Mean squares [Partial]	F ratio	P value
**Model**	**14**	**211.4135**	**15.101**	**49.2852**	**2.11E-08**
**Linear Effects**	**4**	**185.1535**	**46.2884**	**151.072**	**3.75E-10**
**Interaction Effects**	**6**	**18.0454**	**3.0076**	**9.8158**	**0.0005**
**Quadratic Effects**	**4**	**8.2146**	**2.0537**	**6.7026**	**0.0045**
Residual	12	3.6768	0.3064		
Lack of Fit	10	3.5901	0.359	8.2849	0.1124
Pure Error	2	0.0867	0.0433		
Total	26	215.0903

**Table 6:** **Significance of term coefficients for BBD**

Term	Coefficient	Standard error	T value	P value
**Intercept**	**7.7667**	**0.3196**	**24.3025**	**1.42E-11**
**A**	**2.2108**	**0.1598**	**13.8358**	**9.75E-09**
**B**	**-2.4083**	**0.1598**	**-15.0717**	**3.68E-09**
**C**	**1.8517**	**0.1598**	**11.588**	**7.13E-08**
**D**	**-1.1458**	**0.1598**	**-7.1708**	**1.13E-05**
A*B	**-0.9**	**0.2768**	**-3.2518**	**0.0069**
A*C	-0.1675	0.2768	-0.6052	0.5563
A*D	**1.35**	**0.2768**	**4.8778**	**0.0004**
B*C	-0.075	0.2768	-0.271	0.791
B*D	-0.35	0.2768	-1.2646	0.23
C*D	**-1.3125**	**0.2768**	**-4.7423**	**0.0005**
A^2^	0.1996	0.2397	0.8327	0.4213
B^2^	0.3408	0.2397	1.422	0.1805
C^2^	-0.0817	0.2397	-0.3407	0.7392
**D**^**2**^	**1.1346**	**0.2397**	**4.7336**	**0.0005**

**A: Substrate concentration (g/L)B: pH**

**C: Fermentation time (h)D: Na**_**2**_**HPO**_**4**_**Conc. (g/L)**

### Optimization of physical parameters and media components for ethanol production

BBD matrix with response is shown in Table 3. A second order polynomial model fit to the experimental data for optimizing ethanol production via response surface method (RSM) predicts response as a function of four variables and their interactions in terms of their coded values.3

ANOVA calculations listed in Table 5 show that the model F and P values are 49.252 and 2.11 × 10^-8^. This signifies the model with 99% level of confidence (α = 0.01) and all effects namely linear, interaction and quadratic are exhibited. Quality of fit model was estimated by R_adj_^2^ and predicted R^2^ (R_pred_^2^) values were found to be 0.96 and 0.90 respectively which are fairly high and accurate measures of precision ( Ohtani 2000 ). This indicates that only 4% variation in response cannot be suitably explained by the model. Response values for each run calculated by developed model showed little or no variation compared to test results. This indicated that model equation very well corresponded to BBD experimental data. Statistical significance of the model term coefficients was determined by student’s *t*-test and *p* test values as illustrated in Table 6. It was observed that main effects were significant for each of four coded factors whereas interactions among pH and substrate concentration, substrate concentration & Na_2_HPO_4_ concentration, fermentation time & Na_2_HPO_4_ concentration were important as indicated by their high T and low P values.

**Figure 2:**
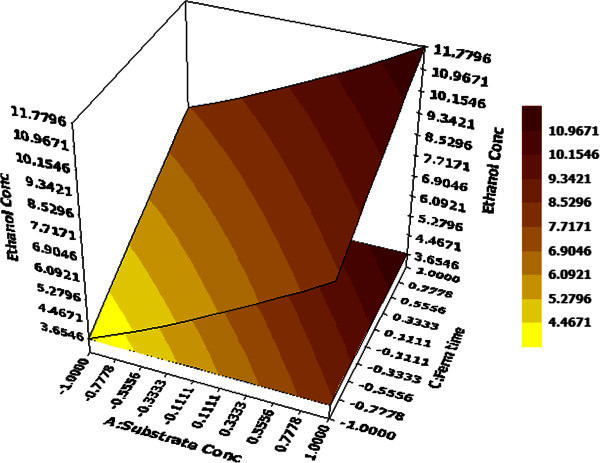
**Effects of substrate concentration and fermentation time on ethanol production hold values: B = 0 (pH = 5.0), D = 0 (Na**_**2**_**HPO**_**4**_**= 0.15 g/L).**

**Figure 3:**
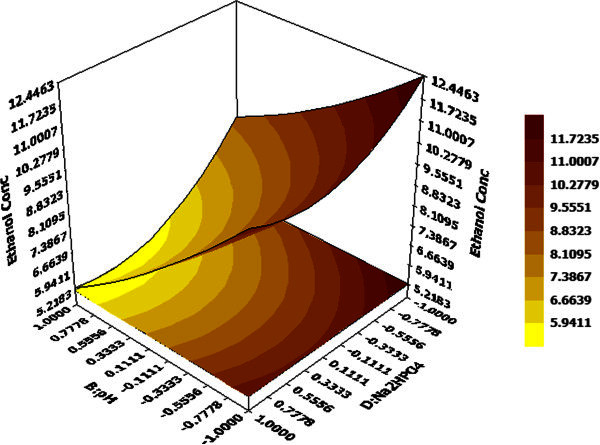
**Effects of pH and Na**_**2**_**HPO**_**4**_**concentration on ethanol production hold values: A =0 (substrate Conc. = 30 g/L), C =0 (fermentation time = 36 h).**

3D response surface graphs display the characteristic effects of key process variables on ethanol concentration. Figure 2 demonstrates the response against substrate concentration and fermentation time while other two factors namely pH and Na_2_HPO_4_ concentration are maintained at their centre point values (0,0), i.e. 5 and 0.30 g/L. The linear surface exhibits a greater first degree effect of both independent variables on system response. An increase in both factors lead to enhanced ethanol yield, maximum being 11.77 g/L at 48 hour and 40 g/L of substrate concentration and decrease in ethanol yield on reduction of the same. Thus, both factors have a positive effect on the dependent variable. On the contrary, effects of Na_2_HPO_4_ concentration and pH at hold values for substrate concentration (30 g/L) and fermentation time (36 h) illustrate that system should be maintained at low values for both variables to attain maximum ethanol production (Figure 3).

**Figure 4:**
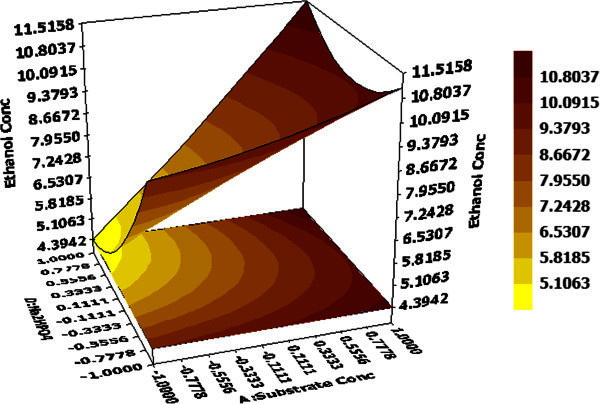
**Effects of substrate concentration and Na**_**2**_**HPO**_**4**_**concentration on ethanol production hold values: B =0 (pH = 5.0), C =0 (fermentation time = 36 hours).**

**Figure 5:**
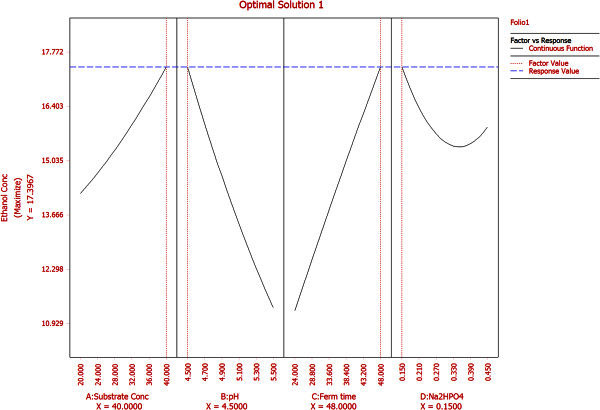
**Optimization conditions for maximizing ethanol yield predicted by reliasoft DOE.**

The surface is more concave nature in this case compared to Figure 2 and represents quadratic and interaction effects in addition to linear ones with maximum ethanol concentration of 12.44 g/L at pH 4.5 and Na_2_HPO_4_ concentration 0.15 g/L. Effect of substrate concentration and Na_2_HPO_4_ concentration on ethanol production at a fixed pH value of 5 and 36 h fermentation time is depicted in Figure 4. It demonstrates that Na_2_HPO_4_ and substrate concentration at their maximum values 0.45 g/L and 40 g/L respectively, lead to maximum ethanol production of 11.51 g/L whereas Na_2_HPO_4_ concentration at its’ lowest value (0.15 g/L) with same substrate concentration yields almost same ethanol (11.15 g/L). Hence, ethanol production is more sensitive to changes in substrate concentration compared to Na_2_HPO_4_concentration when other two variables pH and fermentation time are fixed at their midpoint values. However, interaction effects between these two are + ve and statistically significant as predicted by the model equation for considerable ethanol yield. Based on polynomial model, optimization study was carried out for maximizing ethanol production. Maximum ethanol concentration predicted by the model was found to be 17.39 g/L with 40 g/L (+1) substrate concentration, pH 4.5 (-1), 48 h (+1) fermentation time and 0.15 g/L (-1) Na_2_HPO_4_ (Figure 5). The data was further validated in a shake flask study where the experiment was carried out under optimized condition.

**Table 7:** **Comparative analysis of different ethanol processes with lignocellulosic/waste material**

Sl #	Lignocellulosic/waste raw material	Strain used	Temperature (°C)	pH	Sugar conc. (g/L)	Ethanol conc.(g/L)	Inoculum volume (%v/v)	Yield (% theoretical)	Productivity (g/L/h)	Reference
1	Bagasse pith hydrolysate	*Kluyveromyces* sp. IIPE453	45	4.5	40	17.40	5	88	0.36	**this*paper***
2	Softwood	*Pichiastipitis* CBS6054: *S. cerevisiae* Y5	30	5.0	65	27.40	-	85.1	0.28	( Wan et al. 2012 )
3	Kinnow waste and banana peels	*Consortia of Pachysolentannophilus* (MTCC 1077) and *S. cerevisiae* G	30	-	63	26.84	4:6	83.5	0.55	( Han et al. 2011 )
4	Sugarcane bagasse	*Kluyveromyces fragilis*	35	5.5	180	32.60	-	36	0.45	( Sharma et al. 2007 )
5	Korean food waste	*S. cerevisiae* 7904	35	5.4	75	24.17	2.5	63	0.60	( Sasikumar &Viruthagiri 2008 )
6	Tapioca stem	*Fusarium oxysporum*	30	5.5	33	8.64	2	51.33	0.05	( Man et al. 2010 )
7	Miscanthus biomass	*S. cerevisae*	32	-	140	59.20	7	83.92	1.23	( Magesh et al. 2011 )

### Scale up study in bench scale bioreactor

Scale up study was conducted in bioreactor with optimized conditions yielding 17.44 g/L of ethanol which is almost identical to the model predicted value with residual hexose concentration of 1.2 g/L in the hydrolysate. This validated the accuracy of predicted model and confirmation of an optimum point within system for achieving targeted ethanol production. The ethanol yield (Y_p/S_) in terms of consumed sugar was 88% of theoretical value with specific ethanol productivity of 0.36 g/L/h.

## Conclusion

Identification and optimization of key process variables for ethanol production from SCB pith hydrolysate could successfully be achieved using PBD and RSM. Four variables namely Substrate Conc., pH, fermentation time and Na_2_HPO_4_ were most significant factors affecting ethanol production. Final ethanol concentration and yield attained under optimum fermentation conditions was 17.44 g/L and 88% of theoretical value which was identical to the model predicted response. The ethanol yield, productivity and fermentation conditions for ethanol production from SCB pith via this process was compared with other other lignocellulosic bioethanol processes (Table 7) with different fermenting strains. The ethanol yield obtained with the current process is found to be significantly high in comparison to other processes utilizing different lignocellulosic/waste feedstocks for bioethanol production.
